# Xanthogranulomatous pyelonephritis in a pediatric patient

**DOI:** 10.1590/2175-8239-JBN-2020-0246

**Published:** 2021-04-19

**Authors:** Kamile Eller Gusmão Caixeta, Wallan de Deus Caixeta Matos, Augusto Ventura Ceranto, João Henrique do Amaral e Silva, Kellen Cristina Kamimura Barbosa

**Affiliations:** 1 Universidade Federal do Triângulo Mineiro, Hospital de Clínicas, Pediatria, Uberaba, MG, Brasil.

**Keywords:** Pyelonephritis, Pyelonephritis, Xanthogranulomatous, Stones, coraliform, Kid, Pediatrics, Pielonefrite, Pielonefrite, Xantogranulomatosa, Cálculos, coraliformes, Criança, Pediatria

## Abstract

**Introduction:**

Xanthogranulomatous pyelonephritis consists of a chronic infectious and inflammatory process of the renal parenchyma, a variant of chronic obstructive pyelonephritis. It is more prevalent in middle-aged adults, rare in pediatric patients, with less than 300 cases reported in children worldwide.

**Report:**

Preschooler, aged 2 years and 11 months, male, with 2 months of abdominal distention, increased temperature and intense pallor, associated with microcytic anemia refractory to the use of ferrous sulfate. 1 week before, he had a bulging in his left flank and a hard palpable mass there. Imaging exams (ultrasound and tomography) revealed an overall enlargement of the left kidney, destruction of the renal parenchyma and intense calyceal dilation, forming the "bear's paw" sign, with a staghorn calculus in the pelvis. He underwent treatment with antibiotic therapy and total nephrectomy, with a specimen sent for pathological examination.

**Discussion:**

a disease of uncertain incidence in the pediatric age group, xanthogranulomatous pyelonephritis is more prevalent in male children and affects mainly the left kidney, being frequently associated with the presence of stones. Clinically, it has nonspecific symptoms, the most common being abdominal distension and asthenia. Laboratory exams shows microcytic, leukocytosis, thrombocytosis and increased inflammation, pyuria, hematuria and proteinuria, in addition to bacterial growth in urine culture. The diagnosis is anatomopathological, but it can be hinted by contrasted CT scan, with the classical sign of the "bear's paw". Treatment may include nephrectomy and broad-spectrum antibiotic therapy.

## Resumo

**Introdução:**

A pielonefrite xantogranulomatosa consiste em um processo infeccioso e inflamatório crônico do parênquima renal, variante da pielonefrite obstrutiva crônica. É mais prevalente em adultos na meia-idade, de ocorrência rara em pacientes pediátricos, com menos de 300 casos relatados em pediatria no mundo.

**Relato:**

Pré-escolar de 2 anos e 11 meses, do sexo masculino, com quadro de 2 meses de distensão e aumento da temperatura abdominal e palidez intensa, associado a anemia microcítica refratária ao uso de sulfato ferroso. Apresentava ainda, havia 1 semana da internação, abaulamento em região do flanco esquerdo e massa palpável, de consistência endurecida. Exames de imagem (ultrassonografia e tomografia) revelaram aumento global do rim esquerdo, destruição do parênquima renal e intensa dilatação calicial formando o sinal da "pata de urso", com presença de cálculo coraliforme em pelve. Submetido a tratamento com antibioticoterapia e nefrectomia total, com produto enviado para anatomopatológico.

**Discussão:**

Doença de incidência incerta na faixa pediátrica, a pielonefrite xantogranulomatosa é mais prevalente no sexo masculino na infância e acomete principalmente o rim esquerdo, estando frequentemente associada à presença de cálculos. Clinicamente apresenta-se como quadro arrastado de sintomas inespecíficos, sendo os mais comuns distensão abdominal e astenia. Laboratorialmente, apresenta-se com anemia microcítica, leucocitose, trombocitose e aumento de provas inflamatórias, piúria, hematúria e proteinúria, além de crescimento bacteriano em cultura de urina. O diagnóstico é anatomopatológico, porém pode ser sugerido pela tomografia computadorizada com contraste, que tem como sinal clássico a "pata de urso". O tratamento pode incluir nefrectomia e antibioticoterapia de amplo espectro.

## Introduction

Xanthogranulomatous pyelonephritis consists of a chronic infectious and inflammatory process of the renal parenchyma, a variant of chronic obstructive pyelonephritis^1^. It is an uncommon condition in children, with less than 300 cases in the pediatric population reported in the literature. It can occur at any age, with a higher incidence between the fifth and sixth decades of life^2^. There is almost always urinary tract obstruction associated, especially kidney stones, and it mimics clinically and radiologically important conditions, such as kidney tumors and others infectious processes, such as tuberculosis and renal abscess^1^^-^ 3. Treatment often involves surgery and the diagnosis is based on the histopathological analysis. Suspicion and early diagnosis are essential to prevent mortality and morbidity^1^^,^ 2. In this paper, we describe the clinical, radiological and histological presentation of a pediatric patient with xanthogranulomatous associated with nephrolithiasis, and we discuss the diagnosis and management this condition.

## Report

A 2 year-and-11-month old male preschooler, admitted to the hospital with 2 months of abdominal distension and increased abdominal temperature, accompanied by pallor. In the beginning of the condition, he was brought to medical attention, obtaining a prescription for antiparasitic agents and ferrous sulfate, which had been in use since then, without, however, showing improvement.

**Figure 1:**
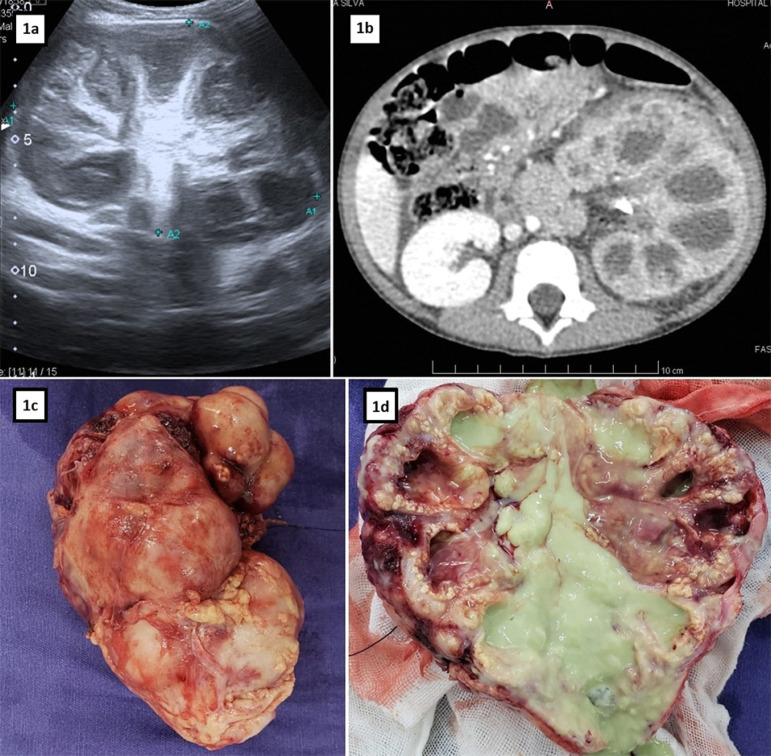
Diagnosis: 1a. Left kidney ultrasound exam. 1b. "Bear's paw" sign on abdominal CT scan. 1c. Macroscopy: left nephrectomy specimen. 1d. Macroscopy: left nephrectomy specimen with longitudinal section.

1 week before hospitalization, there was a bulging in his left flank, when he again sought medical attention. He underwent abdominal ultrasound (Figure 1a), which showed an enlarged left kidney with a globular appearance, with an image suggestive of a staghorn calculus inside.

He was referred to the tertiary service, where, upon admission, he was in regular general condition, pale, tachycardic, with a globose and painful abdomen, with a palpable mass on the left flank and an increased temperature to the touch throughout the abdominal region. Laboratory tests showed leukocytosis (21,420 leukocytes/mm^3^), major microcytic anemia (hemoglobin of 6.3 g/dL and hematocrit of 22.3%), iron deficiency (13.6 mcg/dL), thrombocytosis (757,000 platelets/mm^3^), increased C-reactive protein (16.2 mg/dL) and leukocyturia (28,000 leukocytes/mL). The urine culture showed *Proteus mirabilis* growth. Contrasted computed tomography of the abdomen (Figure 1b) showed a 1.6 cm pelvic staghorn calculus, marked calyceal dilatation and paradoxical pelvic contraction in the left kidney, in addition to diffuse hypodensity and hypoenhancement of the renal parenchyma, which gave the standard image of a "bear paw".

**Figure 2:**
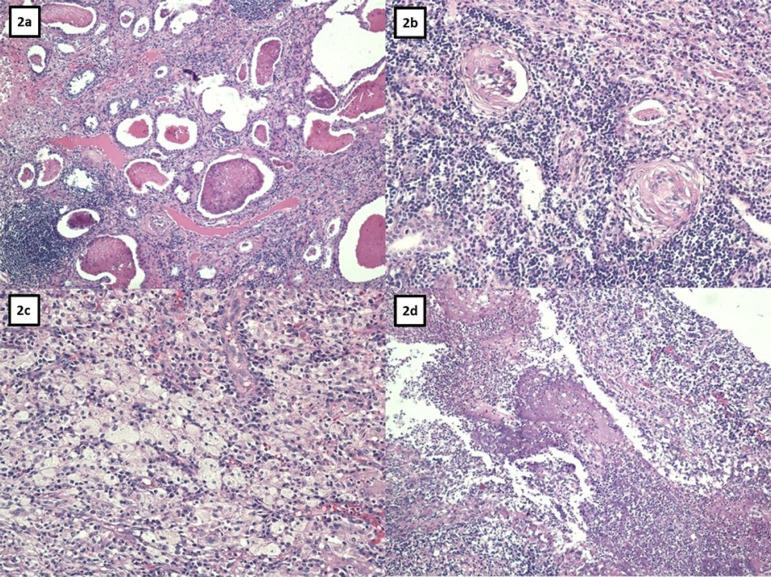
Light microscopy: 2a. Renal parenchyma atrophy. 2b. Glomerular sclerosis. 2c. Xanthogranulomatous infiltrate. 2d. Suppuration.

The patient was then submitted to left nephrectomy by median laparotomy, under the hypothesis of a xanthogranulomatous pyelonephritis. Macroscopically (Figures 1c and 1d), the left kidney was enlarged, with the appearance of pyonephrosis, thick perirenal fat, adhered to the peritoneum, with an intensely dilated pyelocalyceal system and filled with pus. The pathological study showed a kidney with intense pyelocalyceal dilation and a hardened renal parenchyma, with yellow areas. His microscopic examination (Figure 2) revealed extensive productive chronic inflammation with xanthogranulomatous areas coinciding with the destruction of the renal parenchyma, with fibrosis and sclerosis of the remaining glomeruli. The conclusion was xanthogranulomatous pyelonephritis associated with pyonephrosis and urolithiasis with staghorn calculus.

He received 14 days of antibiotic therapy with metronidazole and ceftriaxone and progressed with full recovery, being discharged in good condition, with no report of relapse or functional changes in the contralateral kidney in the year that followed.

## Discussion

Xanthogranulomatous pyelonephritis is chronic inflammation of the renal tissue, characterized by suppuration, renal parenchyma destruction, multinucleated giant cells and lipid-laden macrophages, in addition to inflammatory infiltrate and intense renal fibrosis^1^.

In adults, it is more prevalent in females, in the sixth decade of life, while in pediatric patients, there is a higher prevalence in males, with 60% to 75% of cases occurring in children under 6 years old^4^^,^ 5. The injury is often unilateral, and bilateral involvement is extremely rare. The right kidney is the most frequently involved in adults; however, in children, as in the case described, the left kidney is more frequently involved, according to medical reports^2^.

The condition's etiology is not well understood, but there is a description of factors associated with the development of xanthogranulomatous pyelonephritis, such as: urinary tract obstruction due to lithiasis (30% -50% due to staghorn stones), tumors or urological malformations, ineffective treatment of urinary tract infection, renal ischemia, lipid metabolism and immune response disorders, malnutrition, lymphatic obstruction, vein occlusion and arterial failure^1^^,^ 2 ^,^ 6 ^-^ 8.

Xanthogranulomatous pyelonephritis can be classified according to the extent of the inflammatory process in 3 stages, with stage 1 restricted to the renal parenchyma, stage 2 marked with perirenal fat involvement, and stage 3 with invasion of neighboring structures, such as retroperitoneum, diaphragm and the psoas muscle^3^.

However, the most used classification considers the disease's diffuse or focal presentation. The diffuse form, which is the most common, affects both renal poles and it is characterized by increased renal volume, hydronephrosis, replacement of the corticalmedullary junction by xanthochromic content, and it is often associated with nephrolithiasis^1^^,^ 9. The focal form, which represents less than 15% of the cases, has a pseudotumoral presentation, confined to a segment or renal pole, being more common in the lower pole. Its differential diagnosis include renal tumors (such as Wilms' and renal cell carcinoma) and infectious processes (such as renal tuberculosis and renal abscess)^3^^,^ 10.

Clinically, the disease is subacute or chronic, with nonspecific symptoms, such as anorexia, asthenia, weight loss, intermittent fever without a focus, lumbar or flank pain or tenderness, and may present, on physical examination, a palpable mass in the flank or abdomen. The patient rarely has a history of low urinary symptoms, such as dysuria^1^^,^ 4 ^,^ 8.

Laboratory tests show an increase in inflammatory markers (erythrocyte sedimentation rate and C-reactive protein), leukocytosis, thrombocytosis and microcytic anemia. However, urinalysis can present pyuria, hematuria and proteinuria^2^^,^ 11 ^-^ 13. Urine cultures show growth in only 70% of patients, with *Escherichia coli* and *Proteus mirabilis* being the most frequently found microorganisms^6^.

The most useful imaging tests in the diagnostic process are ultrasound and abdominal computed tomography. In the diffuse form of the disease, ultrasound can present multiple hypoechoic areas, perirenal collection and fatty infiltration of the kidney^1^^,^ 14. In these cases, CT scans reveal a global enlargement of the kidney, with multiple rounded areas of hypoattenuation replacing the renal parenchyma, which represent calyceal dilation and focal areas of renal parenchyma destruction, filled with pus or debris, forming the so-called "bear's paw" sign^15^.There can be extrarenal involvement with perirenal fat stranding, obscuration of the psoas margin and adenopathy. Contrast tomography is useful in planning the surgical procedure, since it enables a better definition of perirenal inflammation. In focal xanthogranulomatous pyelonephritis, ultrasound shows a hypoechoic intrarenal mass and the CT depict a well-defined, hypoattenuating intrarenal mass, with or without perirenal extension^9^^,^ 14 ^,^ 16. In these cases, magnetic resonance imaging can be useful in the absence of hypersignal in the T2 sequence, to differentiate pyelonephritis from tumor masses^1^^,^ 6 ^,^ 16.

Although imaging methods guide and assist the investigation, the definitive diagnosis is histopathological. Macroscopically, the affected kidney presents increased volume and a thickened capsule, with significant loss of renal parenchyma, replaced by nodules of a yellowish tissue, with or without central necrosis. The pelvis and chalices are dilated and filled with stones, debris or pus. Microscopic findings include acute and chronic inflammatory cell infiltrate with multinucleated giant cells and lipid-laden macrophages. It is also possible to identify a slight presence of lymphoid follicles, granulation tissue, in addition to intense fibrosis and hyaline glomerulosclerosis^9^^,^ 17.

Patient management varies in the focal and diffuse forms. In the focal cases, due to the difficult differential diagnosis with kidney tumors, percutaneous ultrasound-guided biopsy or intraoperative biopsy is performed in order to avoid the need for total nephrectomy. Once the diagnosis is confirmed, treatment consists of partial nephrectomy, drainage of pyelonephritis abscess and broad-spectrum antibiotic therapy^2^^,^ 17 ^,^ 18. The diffuse form does not require pre- or intraoperative biopsy. Since more than 80% of cases have non-functional kidneys, total nephrectomy is indicated, which can be difficult to perform and evolve with local complications, due to the extrarenal inflammatory process^17^^,^ 19. The diffuse form has a worse prognosis than the focal one; however, once treated, there are no reports of recurrence in the contralateral kidney^1^.

In the rare cases of bilateral xanthogranulomatous pyelonephritis, management is compromised by the risk of permanent kidney damage. In such cases, partial nephrectomy can be performed; however, conservative treatment is increasingly proposed only with broad-spectrum antibiotic therapy^20^.

In the case presented, we are facing an infrequent, little-known disease, which is associated with nonspecific symptoms without pathognomonic radiological signs, which leads to a late diagnosis. Preoperative diagnosis, especially in the focal form, can be challenging. A high index of clinical suspicion is essential for early diagnosis and treatment that provides a good prognosis. Therefore, xanthogranulomatous pyelonephritis should be included in the differential diagnosis of all patients who have renal or pararenal abscesses, recurrent pyelonephritis or resistance to empirical antibiotic treatment and unilateral renal mass not attributed to other causes, with or without associated nephrolithiasis.
